# Factors Associated with Spontaneous Preterm Birth after Ultrasound-Indicated Cerclage

**DOI:** 10.3390/jpm13121678

**Published:** 2023-12-01

**Authors:** Kyong-No Lee, Sangho Yun, So-Yoon Park, Kyoungseon Kim, Keun-Young Lee, Jae Jun Lee, Ga-Hyun Son

**Affiliations:** 1 Department of Obstetrics and Gynecology, Chungnam National University Hospital, Daejeon 35015, Republic of Korea; kyongnolee@cnuh.co.kr; 2 Institute of New Frontier Research Team, College of Medicine, Hallym University, Chuncheon 24252, Republic of Korea; z4213@daum.net (S.Y.); iloveu59@hallym.or.kr (J.J.L.); 3 Division of Maternal-Fetal Medicine, Department of Obstetrics and Gynecology, Hallym University College of Medicine, Kangnam Sacred Heart Hospital, Seoul 07441, Republic of Korea; sypark@hallym.or.kr (S.-Y.P.); kyoungseonk@hallym.or.kr (K.K.); mfmlee@hallym.ac.kr (K.-Y.L.); 4 Departments of Anesthesiology and Pain Medicine, Hallym University College of Medicine, Chuncheon 24252, Republic of Korea

**Keywords:** cervical insufficiency, obesity, spontaneous preterm birth, ultrasound-indicated cerclage

## Abstract

Ultrasound-indicated cerclage (UIC) is recommended to prevent spontaneous preterm birth (sPTB) in women with a short cervix at mid-trimester and a history of PTB. We assessed the factors related to sPTB after UIC and determined the corresponding risks. This retrospective cohort study was conducted at a university hospital. UIC was performed between 15 and 26 weeks of gestation in women with a cervical length of <2.5 cm. Univariate and multivariate analyses were used to examine factors associated with sPTB after UIC. An earlier gestational age and shorter cervical length at UIC were associated with sPTB after UIC. While PTB history was not associated with an increased risk of sPTB, it did increase the risk of repeat cerclage after UIC. Higher levels of preoperative serum inflammatory markers and obesity significantly increased the risk of sPTB after UIC. These findings provide helpful guidance for patient counseling and management in predicting the delivery timing after UIC in women with a short cervix in the mid-trimester.

## 1. Introduction

Preterm birth (PTB) occurs in approximately 10% of pregnancies and remains the leading cause of neonatal morbidity and mortality worldwide [1,2]. PTB is the leading cause of death in children under 5 years old, accounting for more than one in three of all neonatal deaths. The World Health Organization reported that nearly one million newborns died due to complications associated with PTB in 2020. They also state that three-quarters of these deaths could have been prevented with current, cost-effective interventions [3]. While any birth before 37 weeks of gestation is defined as preterm, births before 28 weeks of pregnancy are defined as “extremely preterm”. Children born extremely preterm have an increased risk of long-term neurodevelopmental disabilities such as cerebral palsy, cognitive disability, and vision and hearing impairments, which ultimately become an important public health problem [4]. With the advancement of neonatal intensive care, the survival rates for neonates born extremely preterm have significantly improved. Currently, the survival rate for neonates born at 28 weeks of gestation is approximately 90%, with >50% of these neonates surviving without any morbidities [4,5,6]. As the survival rate of premature babies born before 28 weeks has increased, not only survival but also long-term complications such as neurodevelopmental disabilities have become important issues. Therefore, predicting pregnancy outcomes in high-risk groups for preterm birth is critical.

A short cervix, defined as less than 2.5 cm upon transvaginal ultrasound at mid-pregnancy, is known to be associated with an increased risk of spontaneous preterm birth (sPTB) [7,8]. Various strategies have been employed to prevent sPTB in women with cervical shortening at mid-trimester. These include the use of progesterone, cerclage, and cervical pessary, along with lifestyle modifications such as smoking cessation, dietary changes, and nutritional supplements. Performing ultrasound-indicated cerclage (UIC) is currently recommended for patients with a short cervical length and a history of PTB. In the largest trial in this field, involving 302 women with a history of sPTB between 16 and 34 weeks and a short cervix (<2.5 cm), it was revealed that cerclage was associated with a significant reduction in perinatal deaths and in births occurring at less than 24 weeks, as well as less than 37 weeks. Additionally, in a meta-analysis covering 504 singleton pregnancies with a history of sPTB before 34 weeks and a short cervix (<2.5 cm) before 24 weeks’ gestation, cerclage was associated with a significant 30% reduction in the risk of PTB before 35 weeks and a 36% reduction in composite perinatal mortality and morbidity [9]. On the other hand, in singleton gestations without prior sPTB but with a cervical length < 2.5 cm in the second trimester, UIC has not shown benefits in preventing preterm delivery or improving neonatal outcomes [10]. However, a recent study demonstrated that in women with an extremely short cervix (<1.0 cm) and no history of sPTB, UIC was associated with a longer time interval to delivery and a lower hazard of earlier delivery after diagnosis compared to patients without UIC [11]. Furthermore, late PTB was less common in patients with UIC compared to those without it. Therefore, UIC may be considered for patients with extreme cervical shortening (<2.5 cm) and no history of sPTB [9,11,12,13,14]. Despite the inherently high risk, certain patients successfully carry pregnancies to term after cerclage placement, while others may face pre- or periviable deliveries. The factors contributing to this variability in clinical outcomes after cerclage placement are not well understood and are likely multifaceted. Several studies have reported that nulliparity, earlier cerclage operation, prior PTB history, advanced cervical dilation, or fetal membranes prolapsing into the vagina are related to the success of physical examination-indicated cerclage [15,16,17]. However, little evidence is available on the progress and prognosis of pregnancy after UIC in patients with a short cervix mid-trimester.

Accordingly, this study aimed to identify the factors associated with the occurrence of sPTB after UIC. Furthermore, examining the factors affecting pregnancy outcomes after UIC may be helpful in considering the use of cerclage in women with a short cervix in the mid-trimester.

## 2. Materials and Methods

**Figure 1:**
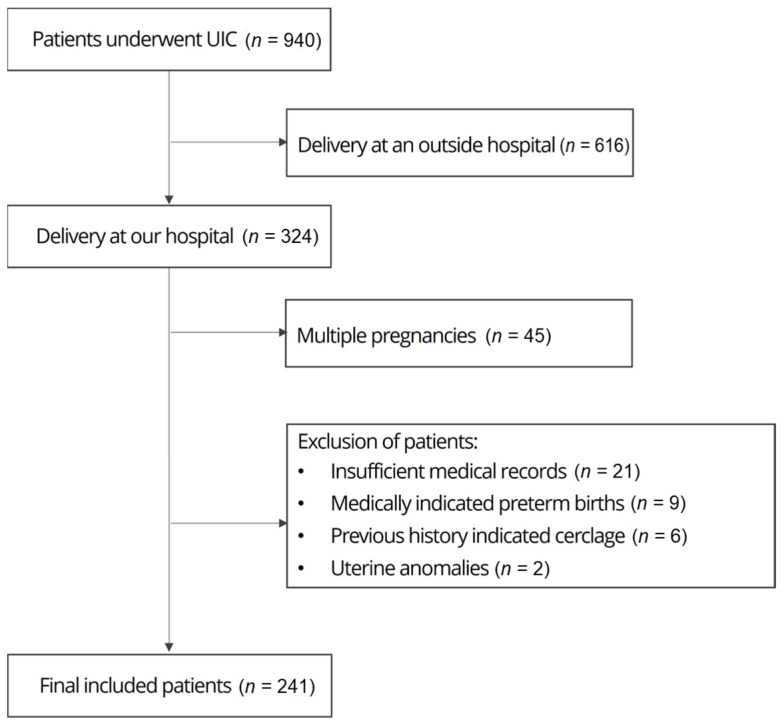
Flow chart of the study.

A total of 940 pregnant women who underwent UIC and delivery between January 2014 and August 2021 at Hallym University Kangnam Sacred Heart Hospital were included in this study. Out of 940 pregnant women, we excluded 616 patients who delivered at other hospitals from the analysis due to the inability to accurately verify information about the delivery. Figure 1 shows the enrollment flowchart. Women who had experienced multiple pregnancies, a previous cervical surgery (including ionization or loop electrosurgical excision procedures), uterine anomalies, previous history-indicated cerclage, or medically indicated PTB were excluded.

Cervical length was measured before UIC via transvaginal ultrasound by placing a probe on the anterior fornix of the vagina while the bladder was empty; pressure was not applied to avoid distorting the cervix. Cervical length was measured from the internal to the external os along the endocervical canal, and the shortest measurement was recorded if dynamic cervical changes were seen. We also measured the width and length of the cervical funneling if present. Maternal blood laboratory tests, including those for serum white blood cell (WBC) count, C-reactive protein (CRP) level, and erythrocyte sedimentation rate (ESR), were performed before UIC. A CRP level > 5.0 mg/L was considered high.

Patient data, including demographic and obstetric history, were extracted from electronic medical records. Maternal height and weight were measured at each outpatient visit and at hospitalization for operation or delivery, and the prepregnancy weight was recorded as stated by the patient. Body mass index (BMI) was defined as the weight in kilograms divided by the square of the height in meters. A prepregnancy BMI ≤ 18.5 kg/m^2^ was defined as underweight, 18.5–24.9 kg/m^2^ as normal weight, 25.0–29.9 kg/m^2^ as overweight, and ≥30 kg/m^2^ as obese.

We performed UIC in patients with a cervical length < 2.5 cm at between 15 and 26 weeks of gestation and without uterine contractions, significant vaginal bleeding, or rupture of the fetal membranes after observation for over 6 h. We performed UIC in patients with a cervical length less than 1 cm, regardless of PTB history. Vaginal progesterone was used in women with no history of PTB and a cervical length between 1 cm and 2.5 cm, and cerclage was performed when cervical shortening progressed during follow-up observation. The McDonald cerclage technique was used in all patients. We used a uniconcave balloon device to prevent the iatrogenic rupture of fetal membranes during cerclage in patients with a ripe, short cervix [18]. Tocolytics were used if uterine contractions were observed on the tocogram postoperatively. All patients received cephalosporin intravenously in the operating room and for 3 days postoperatively. The patients were usually discharged on the third day after cerclage if no other symptoms were present. After discharge, a follow-up was scheduled two weeks later at the outpatient clinic. If no cervical shortening was detected at that time, routine follow-ups were scheduled. Vaginal progesterone (200 mg) was used in all patients after UIC until 36 gestational weeks. We performed repeat cerclage in women whose fetal membrane bulging occurred before 26 weeks of gestation after UIC in the absence of clinical symptoms, including preterm labor, vaginal bleeding, or the rupture of membranes.

Data were analyzed using SPSS version 27.0 (IBM Corp., Armonk, NY, USA). Univariate analysis was performed using Student’s *t*-test, a chi-square test with post hoc tests (custom tables), Fisher’s exact test, the Mann–Whitney U test, or the Kruskal–Wallis test with Dunn’s procedure, as appropriate. All tests were two-tailed, and the variables significantly associated with sPTB in the univariate analysis were further analyzed by multivariate logistic regression. In this regression, we determined the independent association of variables with sPTB adjusted for other variables; a *p*-value < 0.05 was considered statistically significant.

## 3. Results

Of the 241 women included in this study, 138 (57.3%) and 103 (42.7%) experienced preterm and full-term births, respectively. Demographic and clinical characteristics are shown in Table 1. The mean gestational age at UIC was 22.1 ± 3.1 weeks, the mean cervical length was 14.6 ± 5.8 mm, and the mean gestational age at delivery was 33.2 ± 6.3 weeks.

A comparison of the two groups is shown in Table 2. Compared with the full-term delivery group, the preterm delivery group had an earlier gestational age at UIC (preterm: 21.7 ± 3.2 weeks; full-term: 22.7 ± 3.0 weeks; *p* = 0.02), shorter cervical length (preterm: 13.3 ± 5.9 mm; full-term: 16.1 ± 5.6 mm; *p* < 0.001), and higher maternal prepregnancy weight (preterm: 61.7 ± 14.7 kg; full-term: 56.7 ± 9.6 kg; *p* = 0.003) and BMI values (preterm: 23.5 ± 5.0 kg/m^2^; full-term: 21.8 ± 3.6 kg/m^2^; *p* = 0.003). Moreover, we divided patients into underweight *(n* = 30), normal-weight (*n* = 151), overweight (*n* = 38), and obese (*n* = 22) groups according to their prepregnancy BMI and examined the difference in gestational age at delivery according to BMI. As a result, gestational age at delivery was not significantly different between the normal-weight, underweight, and overweight groups but was significantly earlier in the obese group than in the remaining groups (gestational age at delivery: 36.1 (29.2–38.3), 36.9 (32.9–38.4), 35.1 (29.8–38.2), and 32.1 (25.7–35.2), *p* = 0.007, respectively) (Supplementary Table S1). Additionally, preoperative inflammatory serum markers, including WBC count, ESR, and CRP levels, exhibited higher values in the preterm group compared to the full-term group (preterm vs. full-term: 10,004.6 ± 2550.8/μL vs. 9216.4 ± 1949.1/μL; *p* = 0.009, 39.6 ± 18.7 mm/h vs. 31.3 ± 14.0 mm/h; *p* < 0.001, 9.6 ± 12.9 mg/L vs. 5.2 ± 13.4 mg/L; *p* = 0.011, respectively). Furthermore, since prior obstetric history is a crucial factor in determining whether to perform UIC when a short cervix is detected in the mid-trimester, we examined the effect of prior obstetric history on the risk of sPTB after UIC. Of the 241 women, 38 (15.8%) had prior sPTB history, and among them, 27 (11.2%) had a previous history of sPTB occurring before 32 weeks. In contrast, there were 82 (34.0%) patients with a history of full-term delivery. We investigated whether prior PTB history was associated with the occurrence of sPTB after UIC. The proportion of patients with a history of PTB did not differ between the preterm and full-term delivery groups (21/138 [15.2%] vs. 17/103 [16.5%]; *p* = 0.859). However, regarding the frequency of repeat cerclage depending on the presence of prior PTB history, repeat cerclage was performed more frequently in women with prior PTB history than in women without (6/38 [15.8%] vs. 9/203 [4.4%]; *p* = 0.018). Prior PTB history was not associated with sPTB occurrence, even when repeat cerclage was analyzed as a case of sPTB (*p* = 0.593). Therefore, our results suggest that, while prior PTB history was not associated with an increased risk of sPTB after UIC, it did increase the risk of repeat cerclage after UIC. Conversely, having a history of prior full-term delivery was correlated with full-term delivery after UIC (preterm group: 39/138 [28.3%]; full-term group: 43/103 [41.7%]; *p* = 0.039).

### Logistic Regression Analysis

We performed a multivariable logistic regression analysis with gestational age at UIC, cervical length at operation, obesity, prior full-term birth history, and preoperative ESR as the main exploratory variables. In this model, earlier gestational age at operation, short cervical length, high preoperative ESR level, and obesity were independently associated with an increased risk of sPTB after UIC (Table 3).

## 4. Discussion

The results of this study demonstrated that earlier gestational age, shorter cervical length at UIC, preoperative ESR, and maternal prepregnancy obesity were associated with sPTB after UIC. Based on this study’s findings, the risk of sPTB significantly increased with cerclage for earlier gestational age, shorter preoperative cervical length, higher preoperative ESR, or maternal prepregnancy obesity.

Several studies have reported the risk factors related to the success of physical examination-indicated cerclage. Terkildsen et al. examined the factors associated with delivery at >28 gestational weeks after physical examination-indicated cerclage and reported that fetal membranes prolapsing beyond the external cervical os and cerclage operation < 22 weeks were associated with PTB before 28 weeks [19]. In another study, a history of prior second-trimester pregnancy loss, nulliparity, advanced cervical dilation, bulging of the membranes into the vagina, and infection were associated with early preterm delivery before 32 weeks after physical examination-indicated cerclage. Although few studies have assessed factors affecting pregnancy prognosis after UIC, our results were similar to those of previous studies on physical examination-indicated cerclage and demonstrate that longer gestation at UIC and longer cervical length at cerclage were associated with a reduced risk of sPTB [20]. Moreover, our study indicated that increased serum inflammatory markers such as ESR or CRP levels were associated with an increased risk of sPTB after UIC. In addition, maternal obesity is a significant factor in increasing the risk of sPTB after UIC. These findings clinically suggest that in patients with early onset cervical shortening or the presence of prepregnancy maternal obesity, the risk of sPTB after UIC is higher compared to those without these conditions. Therefore, more meticulous follow-up is necessary after cerclage operation. Additionally, high levels of serum preoperative inflammatory markers necessitate a more thorough examination of patient symptoms associated with chorioamnionitis prior to cerclage.

Although a history of PTB was previously considered an important factor in determining treatment options for women with a short cervical length at mid-trimester [21,22,23]. Our findings demonstrate that PTB history did not significantly increase the risk of sPTB after UIC. However, during postoperative follow-up, a greater risk of fetal membrane bulging requiring repeat cerclage was observed in patients with a history of PTB than in those without, which suggests that PTB history can be helpful in screening patients who require urge cerclage operation among patients with cervical shortening. Moreover, women with a history of full-term delivery were more likely to achieve full-term delivery after UIC than those without. This result was consistent with the findings of previous studies stating that patients with a history of full-term delivery who underwent therapeutic cerclage for cervical insufficiency in subsequent pregnancies were more likely to have a higher chance of delivery at ≥35 gestational weeks [24]. However, in our study, pregnant women with a history of full-term delivery exhibited a higher gestational age and longer cervical length at the time of UIC (Supplementary Table S2). Thus, our logistic regression analysis did not show the full-term delivery history associated with pregnancy outcomes after UIC. Our results imply that other factors associated with full-term delivery affect the occurrence of sPTB after UIC.

Inflammation is widely recognized for its significant impact on the occurrence of sPTB, even in cases involving cerclage operations [25,26,27,28]. Previous studies have highlighted the clinical utility of the cytokines and chemokines in amniotic fluid, as mediators of infection/inflammation, in predicting pregnancy outcomes for patients undergoing physical examination-indicated cerclage. Several studies have reported that systemic and local inflammatory markers such as elevated amniotic fluid Interlukein-6, -8, and WBC and low amniotic fluid glucose, in the absence of an intra-amniotic infection, are significantly associated with adverse pregnancy outcomes in patients undergoing physical examination-indicated cerclage [29,30,31]. In another study, it was suggested that various inflammatory, angiogenic, and extracellular matrix-related mediators present in amniotic fluid could serve as biomarkers for predicting sPTB following physical examination-indicated cerclage [32]. However, the necessity for invasive procedures such as second-trimester amniocentesis limits the routine application of these measurements in current clinical practice. Earlier research has shown the simultaneous elevation of multiple inflammatory proteins in both amniotic fluid and systemic circulation [25,33,34]. Li et al. reported that the use of WBC count and neutrophils in combination can help predict histological chorioamnionits, along with preterm premature membrane rupture, after physical examination-indicated cerclage [35]. Nevertheless, the research on inflammation-related biomarkers in plasma samples from patients receiving physical examination-indicated cerclage is insufficient for predicting clinical outcomes. Our findings suggested that an increase in maternal serum inflammatory markers such as CRP and ESR were associated with an increased risk of sPTB after UIC. Presently, there are no established guidelines for the use of progesterone or antibiotics following UIC. Considering maternal serum inflammatory markers may be valuable in refining perioperative management, including the judicious use of antibiotics after cerclage.

Our findings demonstrate that maternal obesity increased the risk of sPTB after UIC. Maternal obesity has been identified as a risk factor for PTB in several studies; two reviews reported that women with obesity grade 2–3 (BMI ≥ 35 kg/m^2^) have an increased risk of very PTB (<32 weeks) and moderate PTB (32–36 weeks) [36,37]. Furthermore, several recent studies have reported an increased risk of extreme PTB (<28 weeks) among women with obesity (BMI ≥ 30 kg/m^2^) [37,38,39,40,41]. Furthermore, previous studies involving women of diverse ethnicities have indicated an association between obesity and extreme PTB [42,43,44]. Shaw et al. conducted a comprehensive analysis of the link between prepregnancy BMI and sPTB in a large population-based cohort. They observed an elevated risk of PTB occurring before 27 weeks in mothers with severe obesity, with this risk varying based on ethnicity and parity [42]. In our study, six patients had obesity grades 2–3, and of these, five (83.3%) delivered preterm. Notably, three of the six patients delivered before 28 weeks, resulting in an extremely preterm birth rate of 50.0%, compared to 19.1% in grade 1 obesity and non-obese patients (grade 1 obesity and non-obese patient vs. grade 2–3 obese, 45/235 vs. 3/6; *p* = 0.095, Fisher’s exact test). Conversely, in other studies, while high obesity increased the risk of preterm premature rupture of membranes and extreme PTB before 28 weeks, it did not correlate with overall PTB [45]. Since sPTB is caused by various factors, and obesity has different effects on each, the effect of obesity should be analyzed in detail to elucidate any direct correlation with PTB or its effects during the gestational weeks of PTB rather than a general analysis.

Few studies have assessed the effect of obesity on PTB in the presence of cervical insufficiency or cervical shortening at mid-trimester. Yalvac et al. reported no differences in latency periods from history-indicated cerclage to delivery according to maternal BMI, suggesting that maternal BMI had no effect on pregnancy outcomes after history-indicated cerclage [46]. In contrast, in a study of 143 mothers receiving cerclage, Poggi et al. reported an inverse correlation between maternal BMI and gestational age after cervical cerclage and stated that a higher BMI was associated with a lower gestational age at delivery after cerclage [47]. In another study involving 148 patients who underwent UIC, BMI was reported not to be a risk factor concerning gestational age at birth [48]. In this study, over half of the study participants were African American (57.5%), and the average BMI was approximately 29 kg/m^2^, contrasting with the clinical characteristics of patients in our study (East Asian, BMI 22.8 kg/m^2^, respectively). These outcomes, being divergent from those of previous studies, suggest that the impact of obesity on the occurrence of preterm birth after UIC may vary depending on factors such as the type of cerclage, patients’ clinical characteristics, and patients’ ethnicity.

Cervical insufficiency is a rare condition that is observed in approximately 1% of pregnant women, limiting large-scale studies on patients undergoing UIC for cervical shortening at mid-trimester [26]. The strength of this study is that it was a relatively large-scale study conducted among patients treated with UIC, analyzing diverse factors affecting the occurrence of sPTB after UIC, including obstetric history and clinical characteristics. Moreover, most patients in this study were Asian and had a normal weight. Previous studies have predominantly examined African American populations with a higher mean BMI. Thus, our study could serve as a reference for pregnancy outcomes after UIC or risk factors for PTB in East Asian women with short cervixes.

This study has several limitations. We only analyzed the data of patients who delivered at the same institution to confirm the birth record clearly. Therefore, the results may differ when including the birth outcomes of individuals who did not give birth at our hospital but at community hospitals or other places. Our institution has a level III neonatal intensive care unit, making it more likely for women with PTB-related symptoms during postoperative follow-up or risk factors related to sPTB to deliver at our institution. Conversely, after UIC, patients without PTB-related symptoms are more likely to deliver in a community hospital; these patients would not have been included in our study, possibly impacting our results. The relatively high incidence of PTB observed in our study compared to other studies may have been caused by this selection bias. Additionally, the cerclage procedures in our study were performed by highly experienced physicians; therefore, the pregnancy outcomes presented in our study may not be reproducible in community hospitals.

## 5. Conclusions

This study demonstrates that earlier gestational age and shorter cervical length at UIC, higher levels of preoperative serum inflammatory markers, and maternal prepregnancy obesity are associated with an increased risk of sPTB after UIC.

This clinically suggests that in cases of early onset cervical shortening or the presence of prepregnancy maternal obesity, more meticulous follow-up after UIC is necessary. These findings provide helpful guidance for patient counseling and management in predicting delivery timing after UIC in women with a short cervix in the mid-trimester. However, since this study was a retrospective study conducted at a single institution, the clinical significance of these risk factors related to sPTB needs to be validated further in large-scale clinical trials.

## Figures and Tables

**Table 1:** Demographic and clinical characteristics of the patients.

Characteristic	Total Patients (*n* = 241)
Age (years)	34.1 ± 4.2
Primipara	128 (53.1)
Preterm birth history	38 (15.8)
Preterm birth < 32 weeks	27 (11.2)
Full-term birth history	82 (34.0)
Height (cm)	161.3 ± 5.3
Prepregnancy body weight (kg)	59.6 ± 13.0
Prepregnancy BMI (kg/m^2^)	22.8 ± 4.5
Hypertensive disease	7 (2.9)
Gestational or overt diabetes	32 (13.3)
Gestational weeks at operation (weeks)	22.1 ± 3.1
Cervical length at operation (mm)	14.6 ± 5.8
Cervical length < 10 mm	65 (27.0)
Presence of cervical funnel	169 (70.1)
Preoperative laboratory results	
ESR (mm/h)	36.4 ± 17.4
CRP (mg/L)	7.7 ± 13.2
WBC count (/μL)	9706 ± 2345
Repeat cerclage	15 (6.2)
Gestational weeks at delivery (weeks)	33.2 ± 6.3
Birthweight (kg)	2.3 ± 1.1

Values are expressed as mean ± SD or n (%). BMI, body mass index; ESR, erythrocyte sedimentation rate; CRP, C-reactive protein; WBC, white blood cell.

**Table 2:** Comparison of clinical characteristics between the preterm delivery group and full-term delivery group.

Characteristic	Preterm Group (*n* = 138)	Full-Term Group (*n* = 103)	*p* Value
Age (years)	33.9 ± 4.2	34.3 ± 4.1	0.508
Primipara	80 (58.0)	48 (46.6)	0.118
Preterm birth history	21 (15.2)	17 (16.5)	0.859
Full-term birth history	39 (28.3)	43 (41.7)	0.039
Height (cm)	161.5 ± 5.4	161.1 ± 5.3	0.602
Prepregnancy body weight (kg)	61.7 ± 14.7	56.7 ± 9.6	0.003
Prepregnancy BMI (kg/m^2^)	23.5 ± 5.0	21.8 ± 3.6	0.003
Gestational weeks at operation (weeks)	21.7 ± 3.2	22.7 ± 3.0	0.020
Cervical length at operation (mm)	13.3 ± 5.9	16.1 ± 5.6	<0.001
Cervical length < 10 mm	50 (36.2)	15 (14.6)	<0.001
Presence of cervical funnel	104 (75.4)	65 (63.1)	0.047
Preoperative laboratory results			
ESR (mm/h)	39.6 ± 18.7	31.3 ± 14.0	<0.001
CRP (mg/L)	9.6 ± 12.9	5.2 ± 13.4	0.011
WBC count (/μL)	10,004.6 ± 2550.8	9216.4 ± 1949.1	0.009
Repeat cerclage	12 (8.7)	3 (2.9)	0.103
Gestational weeks at delivery (weeks)	29.4 ± 5.8	38.5 ± 1.0	<0.001
Birthweight (kg)	1.6 ± 0.9	3.2 ± 0.4	<0.001

Values are expressed as mean ± SD or *n* (%). BMI, body mass index; ESR, erythrocyte sedimentation rate; CRP, C-reactive protein; WBC, white blood cell.

**Table 3:** Risk of spontaneous preterm birth after ultrasound-indicated cerclage.

Variable	Odds Ratio	95% CI	*p* Value
Gestational weeks at operation (weeks)	0.897	0.817–0.985	0.023
Cervical length at operation (mm)	0.915	0.870–0.963	0.001
Obesity	4.067	1.081–15.305	0.038
Preoperative ESR (mm/h)	1.030	1.011–1.049	0.002
Full-term birth history	1.256	0.689–2.288	0.457

CI, confidence interval; ESR, erythrocyte sedimentation rate.

## Data Availability

The data presented in this study are available from the corresponding author upon request. The data are not publicly available due to patients’ privacy.

## Supplementary Materials

The following supporting information can be downloaded at: https://www.mdpi.com/article/10.3390/jpm13121678/s1, Supplementary Table S1: Comparison of clinical characteristics between groups divided according to prepregnancy BMI; Supplementary Table S2: Comparison of patients’ characteristics according to full-term delivery history.
