# Supplementary material for: Factors Associated with Spontaneous Preterm Birth after Ultrasound-Indicated Cerclage
Source: J Pers Med. 2023 Dec 1;13(12):1678. doi: 10.3390/jpm13121678 (PMC10744759; doi:10.3390/jpm13121678)
Supplement: Supplementary file 1 [file jpm-13-01678-s001.zip › jpm-2722340-supplementary.pdf]

Supplementary Table S1. Comparison of clinical characteristics between groups divided according to pre-pregnancy BMI

	Underweight (n=30)	Normal (n=151)	Overweight (n=38)	Obese (n=22)	P value
Age	34.0 (31.3-36.0)	34.0 (31.3-37.0)	34.0 (30.1-38.0)	35.0 (30.8-37.3)	0.952
Pre-pregnancy body weight (kg)	45.5 (43.0-48.0)	54.0 (52.0-60.0)[a]	68.0 (65.0-75.0)[b]	85.0 (80.0-95.0)[c,d]	<0.0001
Pre-pregnancy BMI (kg/m^2)	17.7 (17.2-18.0)	21.1 (19.9-22.7)[a]	26.4 (25.5-27.7)[b]	32.9 (30.8-35.0)[c,d]	<0.0001
Gestational weeks at operation (weeks)	22.6 (21.1-24.9)	22.1 (19.4-24.4)	21.4 (19.4-24.1)	22.9 (19.3-25.6)	0.231
Cervical length at operation (mm)	16.2 (12.4-19.6)	14.7 (9.7-19.7)	14.7 (9.8-17.4)	11.4 (7.1-19.0)	0.491
Cervical length <10 mm	7 (23.3)	39 (25.8)	11 (28.9)	8 (36.4)	0.714
Gestational weeks at delivery (weeks)	36.9 (32.9-38.4)	36.1 (29.2-38.3)	35.1 (29.8-38.2)	32.1 (25.7-35.2)[c,d]	0.007
Preterm birth	14 (46.7)	82 (54.3)	23 (60.5)	19 (86.4)[c]	0.021
Birthweight (kg)	2.8 (1.9-3.0)	2.6 (1.4-3.1)	2.3 (1.4-2.9)	2.3 (0.8-2.7)	0.089

BMI, body mass index; ESR, erythrocyte sedimentation rate; CRP, C-reactive protein; WBC, white blood cell Values are expressed as median(interquartile range) or n (%).

a: significantly different between underweight and normal (p<0.05)

b: significantly different between underweight or normal and overweight (p<0.05)

c: significantly different between underweight or normal and obese (p<0.05)

d: significantly different between overweight and obese (p<0.05)

Supplementary Table S2. Comparison of patients' characteristics according to full-term delivery history.

	Presence of full-term delivery history (n=83)	Absence of full-term delivery history (n=157)	P value
Age (years)	35.8±3.4	33.2±4.3	<0.0001
Height (cm)	161.9±5.0	161.1±5.5	0.274
Pre-pregnancy body weight (kg)	57.8±11.3	60.6±13.8	0.092
Pre-pregnancy BMI (kg/m^2)	22.0±3.9	23.2±4.8	0.039
Gestational weeks at operation (weeks)	22.8±3.1	21.8±3.1	0.021
Cervical length at operation (mm)	15.9±5.5	13.8±6.0	0.007
Cervical length <10 mm	14 (16.9)	51 (32.3)	0.014
Presence of cervical Funnel	54 (65.1)	115 (72.8)	0.237
Preoperative laboratory results			
ESR (mm/hr)	33.3±14.1	37.5±18.6	0.054
CRP (mg/L)	8.3±16.4	7.4±11.4	0.636
WBC count (/µL)	9303.1±2182.8	9859.1±2405.0	0.080
Repeat cerclage	4 (4.8)	11 (7.0)	0.588
Gestational weeks at delivery (weeks)	34.1±6.4	32.9±6.3	0.152
Birthweight (kg)	2.4±1.1	2.2±1.1	0.116

BMI, body mass index; ESR, erythrocyte sedimentation rate; CRP, C-reactive protein; WBC, white blood cell Values are expressed as mean±SD or n (%).
